# Tomato receptor-like cytoplasmic kinase Fir1 interacts with a negative regulator of jasmonic acid signaling

**DOI:** 10.17912/micropub.biology.000736

**Published:** 2023-02-25

**Authors:** Guy Sobol, Gregory B. Martin, Guido Sessa

**Affiliations:** 1 School of Plant Sciences and Food Security, The George S. Wise Faculty of Life Sciences, Tel Aviv University, Tel Aviv, Israel; 2 Boyce Thompson Institute for Plant Research, Ithaca NY, USA; 3 Plant Pathology and Plant-Microbe Biology Section, School of Integrative Plant Science, Cornell University, Ithaca NY, USA

## Abstract

Plant cells detect potential pathogens through plasma membrane-localized pattern recognition receptors (PRRs) that recognize microbe-associated molecular patterns (MAMPs) and activate pattern-triggered immunity (PTI). PRR-mediated MAMP perception is linked to PTI signaling by receptor-like cytoplasmic kinases (RLCKs). In tomato, Flagellin-sensing 2 (Fls2)/Fls3 interacting RLCK 1 (Fir1) is involved in PTI triggered by flagellin perception. Fir1 is necessary for regulation of jasmonic acid (JA) signaling and is involved in pre-invasion immunity. We show that Fir1 physically interacts with JASMONATE-ZIM-DOMAIN PROTEIN 3 (JAZ3), a negative regulator of JA signaling. This finding suggests that Fir1 modulates JA signaling by regulating JAZ3.

**Figure 1. Fir1 interacts with JAZ3 in planta and in vitro:**
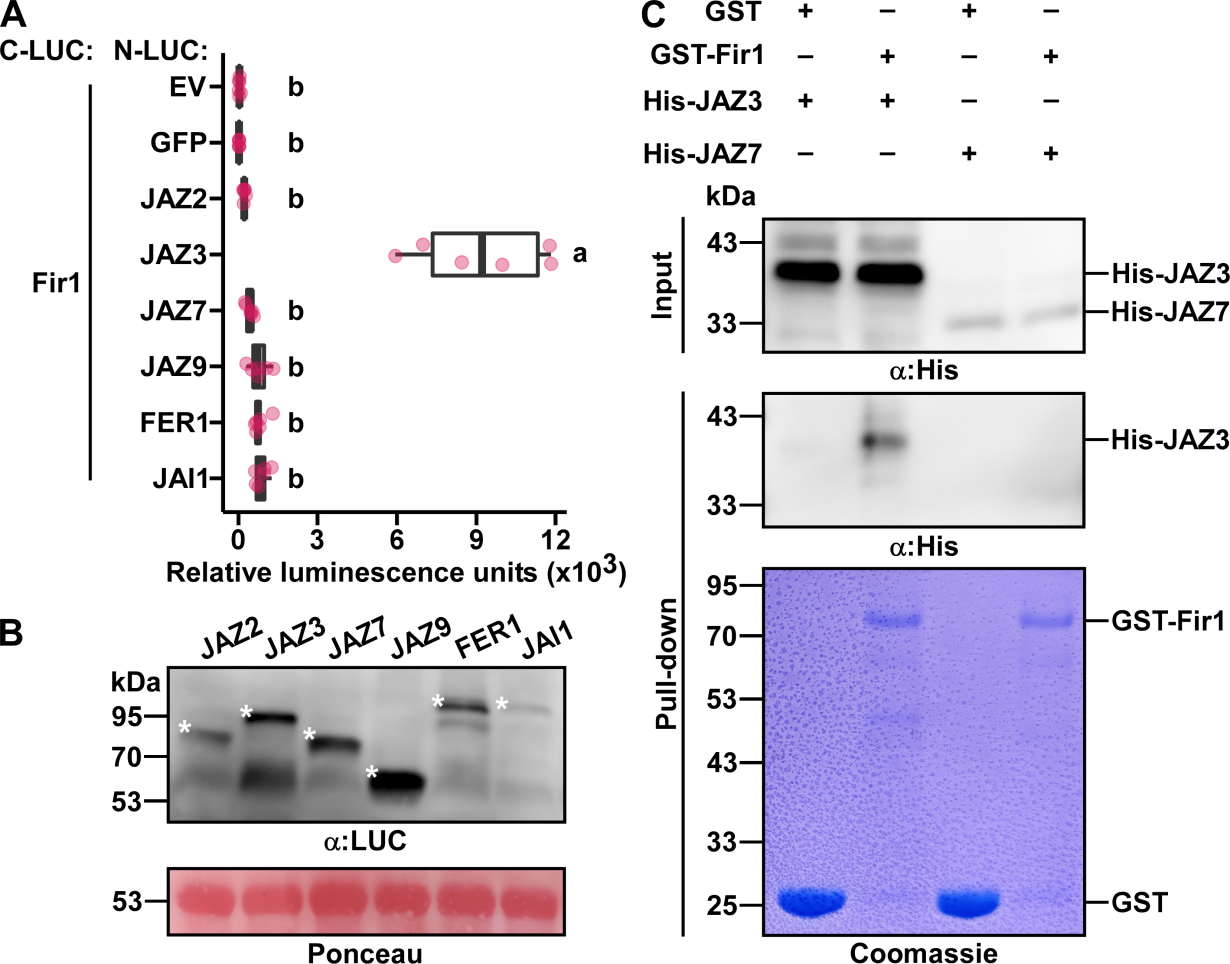
(A) The indicated proteins were fused to C-LUC or N-LUC and co-expressed via *A. tumefaciens* in *N. benthamiana* leaves. Empty vector (EV) or green fluorescent protein (GFP) were used as negative controls. Luciferase activity was quantified by measuring relative luminescence at 48 h after agro-infiltration. The experiment was repeated three times with similar results. Horizontal lines inside boxes represent median values. Median values are indicated by bold lines inside each box. The third and first quartiles of the value distribution are represented by the top and bottom sides of the boxes, respectively. The extreme values within 1.5 times the interquartile range are shown by lines extending from the boxes. Individual data points are shown as circles, and letters denote statistical significance as assessed by one-way ANOVA and Tukey's post-hoc test ( *P* < 0.05). (B) Expression of the indicated proteins fused to the N-terminal half (N-LUC) of the luciferase protein in leaves of *N. benthamiana* plants. Proteins were detected by immunoblot analysis using anti-luciferase (α:LUC) antibodies. Ponceau S staining of Rubisco is shown as a loading control. Asterisks denote bands of the expected molecular weight. (C) GST or GST-Fir1 bound to glutathione resin was added to lysates of *E. coli* cells expressing His-JAZ3 or His-JAZ7, and incubated overnight. Proteins eluted from the glutathione resin were fractionated by SDS-PAGE and subjected to Western blot analysis with anti-His (α:His) antibodies or stained with Coomassie Brilliant Blue, as indicated. The experiment was repeated four times with similar results. In A, B and C, results of a representative experiment are shown.

## Description

The plant immune system is equipped with plasma membrane-localized pattern recognition receptors (PRRs) that mediate early detection of potential pathogens by recognizing conserved microbe-associated molecular patterns (MAMPs) (DeFalco and Zipfel 2021). In tomato plants ( *Solanum lycopersicum* ), the PRR repertoire includes the Flagellin sensing 2 (Fls2) (Robatzek et al. 2007) and Flagellin sensing 3 (Fls3) (Hind et al. 2016) receptors, which recognize the bacterial flagellin-derived peptides flg22 and flgII-28, respectively. Binding of flg22 and flgII-28 by Fls2 and Fls3 triggers signaling pathways that initiate a battery of immune responses, known as pattern-triggered immunity (PTI) (Liang and Zhou 2018). The output of PTI often consists of generation of reactive oxygen species (ROS), gene transcription reprogramming, activation of mitogen-activated protein kinases (MAPKs), closure of stomata, and the accumulation of antimicrobial proteins (DeFalco and Zipfel 2021).

In several instances, receptor-like cytoplasmic kinases (RLCKs) have been found to link PRR-mediated MAMP perception to downstream signaling pathways (Liang and Zhou 2018). RLCKs interact with and modulate the activity of a variety of proteins that participate in branches of PTI signaling. For example, activity of the Arabidopsis ( *Arabidopsis thaliana* ) RESPIRATORY BURST OXIDASE HOMOLOG D (RBOHD), which is involved in ROS production, is modulated through phosphorylation by several RLCKs, including RPM1-INDUCED PROTEIN KINASE (RIPK) (Li et al. 2021), BOTRYTIS-INDUCED KINASE1 (BIK1) (Kadota et al. 2014; Li et al. 2014), and avrPphB SUSCEPTIBLE 1-LIKE 13 (PBL13) (Lee et al. 2020). The ion channels SLOW ANION CHANNEL-ASSOCIATED 1 HOMOLOG 3 (SLAH3) and REDUCED-HYPEROSMOLALITY-INDUCED-[Ca ^2+^ ] INCREASE 1.3 (OSCA1.3), are directly phosphorylated by avrPphB SUSCEPTIBLE 1-LIKE 27 (PBL27) and BIK1, respectively, to generate an ion flow that leads to stomatal closure (Liu et al. 2019; Thor et al. 2020). Immunity-associated MAPKs serve as direct substrates of several RLCKs, including PBL27 (Yamada et al. 2016), OsRLCK185 (Wang et al. 2017), BRASSINOSTEROID-SIGNALING KINASE 1 (BSK1) (Yan et al. 2018; Shi et al. 2022), and members of the RLCK subfamily VII (Bi et al. 2018). Finally, BIK1 directly interacts in the nucleus with WRKY transcription factors to modulate gene expression associated with the jasmonic acid (JA) and salicylic acid (SA) defense hormones (Lal et al. 2018).

We recently investigated the role of the tomato Fls2/3 interacting RLCK 1 (Fir1) in PTI (Sobol et al. 2022). Fir1 was found to interact with Fls2 and Fls3, suggesting that it plays a role as an early signaling component of PTI activated by flagellin perception. Phenotypic analyses revealed that a loss-of-function mutation of the *fir1* gene causes increased susceptibility to the bacterial pathogen *Pseudomonas syringae* pv. *tomato* ( *Pst* ) DC3000. In addition, Fir1 appears to participate in pre-invasion immunity and stomatal closure. Consistent with its interaction with Fls2 and Fls3, Fir1 is required for flagellin-induced ROS production and *PATHOGENESIS-RELATED 1b* mRNA expression. Analysis of gene expression profiles of *fir1* mutant plants upon PTI activation revealed a deregulated JA response. In line with this finding, *fir1* loss-of-function mutations diminished the contribution of coronatine to virulence of *Pst* bacteria, and enhanced resistance to *Botrytis cinerea* , a necrotrophic fungus, after activation of PTI. These findings suggest that Fir1 is necessary for the appropriate activation of PTI and support a model in which Fir1 regulates JA signaling to inhibit a JA response (Thaler et al. 2012).

We hypothesized that Fir1 modulates the JA response by interacting with components of the JA signaling pathway. To assess this hypothesis, a split-luciferase complementation assay was employed to test the interaction between Fir1 and members of the JASMONATE-ZIM-DOMAIN (JAZ) protein family, which negatively regulate JA signaling (Pauwels and Goossens 2011), and whose transcripts were differentially expressed in *fir1-1* mutant plants during activation of PTI (JAZ2, JAZ3, JAZ7, and JAZ9) (Sobol et al. 2022). In addition, we tested the interaction of Fir1 and the JA receptor JASMONIC ACID–INSENSITIVE1 (JAI1) (Li et al. 2004), and FER1 (homolog of Arabidopsis FERONIA), which inhibits coronatine-induced signaling to promote disease resistance (Guo et al. 2018). In these experiments, Fir1 was fused to the C-terminal fragment of the luciferase protein (C-LUC) and co-expressed via *Agrobacterium* *tumefaciens* in *Nicotiana benthamiana* leaves with a candidate interactor fused to the N-terminal fragment of luciferase (N-LUC). As negative controls, C-LUC-Fir1 was co-expressed with either an empty N-LUC vector or with N-LUC fused to the green fluorescent protein (GFP). The luminescence emitted from leaf discs was measured 48 hours after agro-infiltration and used to quantify interactions. Leaf disks co-expressing Fir1 and JAZ3, but not any of the other protein combinations, emitted a significantly higher luminescence than the negative controls (Figure 1A). Western blot analysis was used to validate that each fusion protein was expressed in the inoculated plant tissues (Figure 1B and Roberts et al. 2019).

We used an *in vitro* pull-down assay to confirm the interaction between Fir1 and JAZ3. Fir1 fused to the glutathione- *S* -transferase (GST) protein was expressed in *E. coli* and purified. Lysates of bacterial cells expressing His-tagged JAZ3 and JAZ7 (negative control) were incubated with immobilized GST-Fir1 or GST (negative control) and washed. Western blot analysis with anti-His antibodies detected His-JAZ3, but not His-JAZ7 among the proteins pulled-down by GST-Fir1 (Figure 1C), suggesting a direct interaction between Fir1 and JAZ3.

JAZ3 is a negative regulator of JA signaling (Pauwels and Goossens 2011) and it has been shown to be targeted by pathogen effector proteins (Yang et al. 2017; Anderson et al. 2019). For example, the *Pst* type III secreted effector HopBB1 interacts with the TCP14 transcription factor, a repressor of a subset of JA responses. To activate JA response genes, and consequently increase pathogen virulence, HopBB1 reduces TCP14 abundance by coupling it with JAZ3, which sends it to degradation in the SCF ^COI^ degradation complex (Yang et al. 2017). The HaRxL10 effector protein from the oomycete pathogen *Hyaloperonospora arabidopsidis* ( *Hpa* ) targets JAZ3 to decrease its abundance. The reduction in JAZ3 abundance activates JA responses, and suppresses SA responses, which normally restrict *Hpa* growth (Anderson et al. 2019). Together, the results indicate that Fir1 interacts in planta and *in vitro* with JAZ3, which represents a candidate signaling component regulated by Fir1.

## Methods

Bacterial strains, plant materials, and growth conditions

*Nicotiana benthamiana* plants (Goodin et al. 2008) were cultivated at 25°C in a phytochamber under long-day conditions (16 h/8 h, light/dark). The bacterial strains used were: *Escherichia coli* DH5a (Invitrogen), *E. coli* Rosetta (Novagen), and *Agrobacterium tumefaciens* GV2260 (Deblaere et al. 1985). *E. coli* was grown in Lysogeny broth (LB) medium at 37°C and *A. tumefaciens* in LB medium at 30°C with the required antibiotics.

Split-luciferase complementation assay

Genes encoding JAZ2, JAZ3, JAZ7, JAZ9, FER1, and JAI1 were PCR-amplified from tomato cDNA with primers 1-2 (JAZ2), 3-4 (JAZ3), 5-6 (JAZ7), 7-8 (JAZ9), 9-10 (FER1), or 11-12 (JAI1) (Table 1), and cloned in frame with N-LUC in the pCAMBIA1300:N-LUC plasmid (Chen et al. 2008). pCAMBIA1300:N-LUC carrying a GFP-N-LUC fusion and pCAMBIA1300:C-LUC carrying a C-LUC-Fir1 fusion were prepared as described by Sobol et al. (2022). The obtained vectors were transformed into *A. tumefaciens* GV2260 bacteria, which were infiltrated into *N. benthamiana* leaves for protein co-expression. Split-luciferase complementation assays were carried out as described (Majhi et al. 2019).

*In vitro* pull-down assay

Tomato cDNA was used to PCR-amplify genes encoding JAZ3 and JAZ7 using primers 15-16 (JAZ3), or 17-18 (JAZ7) (Table 1). JAZ3 and JAZ7 were cloned downstream and in frame to a 10xHis-tag in the pET-16b vector (Novagen). The pGEX-4T1 vector carrying Fir1 fused to GST (GST-Fir1) was prepared as described by Sobol et al. (2022). Plasmids carrying His-JAZ3, His-JAZ7, GST, and GST-Fir1 were transformed into the Rosetta *E. coli* strain and *in vitro* pull-down assays were carried out as described (Sobol et al. 2022).

Accession numbers

Sequence data used in this article can be located in the Solanaceae Genomics Network database ( https://solgenomics.net/ ) under the following accession numbers: *Fir1* (Solyc12g099830), *JAZ2* (Solyc12g009220), *JAZ3* (Solyc03g122190), *JAZ7* (Solyc11g011030), *JAZ9* (Solyc08g036640), *FER1* (Solyc09g015830), *JAI1* (Solyc05g052620).

## Reagents

**Table 1.** Commercial products used in this study.

**Table:** 

**Product**	**Catalog number**	**Company**
Anti-Luciferase antibody produced in rabbit	L0159	Sigma-Aldrich
D-Luciferin sodium salt	L6882	Sigma-Aldrich
Monoclonal Anti-polyHistidine antibody produced in mouse	H1029	Sigma-Aldrich
Glutathione resin	L00206	GenScript

**Table 2.** Primers used in this study.

**Table:** 

**Primer ID**	**Name**	**Sequence (5’ to 3’)**	**Purpose**
1	JAZ2_F_SacI	AAAAAAGAGCTCATGGGGTCATCGGAAAATATGGATTC	Cloning of *JAZ2* into pCAMBIA:N-LUC
2	JAZ2_R_SalI	AAAAAAGTCGACGAAATATTGCTCAGTTTTAACAAATTGAGCACC	
3	JAZ3_F_KpnI	AAAAAAGTTACCATGTCGAATTTATGTGACGCTCGC	Cloning of *JAZ3* into pCAMBIA:N-LUC
4	JAZ3_R_SalI	AAAAAAGTCGACTAACTTGAAATTGAGATCGAGCTGATCTTC	
5	JAZ7_F_SacI	AAAAAAGAGCTCATGGATTCAAGAATGGAGATAGATTTTATGGAC	Cloning of *JAZ7* into pCAMBIA:N-LUC
6	JAZ7_R_SalI	AAAAAAGTCGACGTTTTCCCAATGAACGCTTGACG	
7	JAZ9_F_KpnI	AAAAAAGGTACCATGAGAAGAAATTGTAATTTGGAGCTCACTC	Cloning of *JAZ9* into pCAMBIA:N-LUC
8	JAZ9_R_SalI	AAAAAAGTCGACTTTGTGATATGGCGAAGTTGCTTGAAC	
9	FER1_F_SacI	AAAAAAGAGCTCATGTTTAGGTCATGGGGTGACG	Cloning of *FER1* into pCAMBIA:N-LUC
10	FER1_R_SalI	AAAAAAGTCGACACGTCCTTTTGGATTCATGATTTGAGAG	
11	JAI1_F_SacI	AAAAAAGAGCTCATGGAGGAACGGAACTCAACG	Cloning of *JAI1* into pCAMBIA:N-LUC
12	JAI1_R_SalI	AAAAAAGTCGACTTCAGCGAGAAGGTAAGTTGGGTC	
13	pET-16b_F	GATATGGCCGCTGCTGTG	Cloning of *JAZ3* into pET-16b
14	pET-16b_R	AGCAATAACTAGCATAACCCCTTG	
15	JAZ3_F	CACAGCAGCGGCCATATCTCGAATTTATGTGACGCTCGC	
16	JAZ3_R	CAAGGGGTTATGCTAGTTATTGCTCTATAACTTGAAATTGAGATCGAGCTG	
17	JAZ7_F_BamHI	AAAAAAGGATCCTATGGATTCAAGAATGGAGATAGATTTTATGGAC	Cloning of *JAZ7* into pET-16b
18	JAZ7_R_BamHI	AAAAAAGGATCCTTAGTTTTCCCAATGAACGCTTGACG	

**Table 3.** Plasmids used in this study

**Table:** 

**Plasmid**	**Description**	**Reference**
pCAMBIA1300:C-LUC	Split-luciferase complementation assay	Chen et al., 2008
pCAMBIA1300:C-LUC-Fir1		Sobol et al., 2022
pCAMBIA1300:N-LUC		Chen et al., 2008
pCAMBIA1300:GFP-N-LUC		Majhi et al., 2019
pCAMBIA1300:JAZ2-N-LUC		This study
pCAMBIA1300:JAZ3-N-LUC		This study
pCAMBIA1300:JAZ7-N-LUC		This study
pCAMBIA1300:JAZ9-N-LUC		This study
pCAMBIA1300:FER1-N-LUC		This study
pCAMBIA1300:JAI1-N-LUC		This study
pET-16b	*In vitro* pull-down assay	Novagen
pET-16b:His-JAZ3		This study
pET-16b:His-JAZ7		This study
pGEX-4T1		GE Healthcare
pGEX-4T1:Fir1		Sobol et al., 2022
